# Supplementary material for: Network location and clustering of genetic mutations determine chronicity in a stylized model of genetic diseases
Source: Sci Rep. 2022 Nov 19;12:19906. doi: 10.1038/s41598-022-23775-9 (PMC9675813; doi:10.1038/s41598-022-23775-9)
Supplement: Supplementary file 1 — Supplementary Information. [file 41598_2022_23775_MOESM1_ESM.pdf]

Appendix: Network location and clustering of genetic mutations determine chronicity in a stylized model of genetic diseases

Piotr Nyczka[1,2], **Johannes Falk**[2,*], **and Marc-Thorsten Hütt**[2]

[1]Faculty of Management, Wrocław University of Science and Technology
[2]Department of Life Sciences and Chemistry, Jacobs University, D-28759 Bremen, Germany
[*]j.falk@jacobs-university.de

Evaluation of the parameter a

In order to provide some intuition on the range of values for the percentage a of logical ANDs – which is one of the key parameters of our model – we resort to genome-scale metabolic models. Here we employ two strategies to estimate the value of a from such metabolic reconstructions.

The first method counts the Boolean operations in the gene-to-reaction mappings within genome-scale metabolic models and computes the ratio

$$a = \mathrm{AND}/(\mathrm{AND}+\mathrm{OR}). \tag{1}$$

This yields – depending on the model – a between 0.105 and 0.15 (see Table 1). The analysis was performed with the COBRA package for Python (Cobra version 0.19.0; Python version 3.6).

Table 1. Estimation results for the parameter a for three genome-scale metabolic models of human cells, Recon 1[1], Recon 2[2] and Recon 3D[3].

Estimation of a			
Method	Recon 1	Recon 2.3	Recon 3D
1 (gene-reaction associations)	0.15	0.129	0.105
2 (complex reactions)	0.24	0.08	0.21

The second method evaluates each (reaction) node together with its next-to-nearest neighbours. In this sense, the method is closer to the nature of nodes in our model, which do not represent individual reactions, but rather more complex regulatory entities, summarizing metabolic flow and genetic control. Such subgraph objects may behave like AND or OR gate depending on the mutual proportions of the alternative sources and the number of reactants.

This method utilizes information about reaction network including their directionality. Each reaction has a form of $R_1 + R_2 + ... \to P_1 + P_2 + ...$, where R_i are reactants and P_j products. Our quantification focuses on reactants alone. Each reactant may have one or more possible sources, where the number of these sources for each reactant is k_i. Hence, the reaction itself corresponds to a Boolean AND, but alternative sourcing is an analogue of a Boolean OR. Such a subgraph has the structure of multiple logical ORs fed to an single logical AND: $(R_{1,1}|R_{1,2}|...) + (R_{2,1}|R_{2,2}|...) + ... \to P_1 + P_2 + ...$. In order to estimate the parameter a, we need to convert this whole entity to just a single AND or OR.

All fluxes are regarded to be discrete, hence inputs can be characterized by a probability c_{in} of having reactant i from the source j, $R_{i,j}$. With this quantity, the probability of reaction taking place, c_{out}, can be computed. For a logical AND $c_{out} < c_{in}$ whereas for a logical OR $c_{out} > c_{in}$, there are also special cases of $c_{in} = 0$ and $c_{in} = 1$. The same classification can be done for more complicated structures, like the one discussed above. For simple gates, there is always (for each c_{in}) either $c_{out} < c_{in}$ or $c_{out} > c_{in}$ depending on the gate. However, for more complicated entities this inequality may change sign upon change of c_{in}.

Way to overcome this difficulty is to compare whole range of $c_{in} \in [0,1]$ by taking integral and then comparing:

$$c_{out} = \prod_i (1-(1-c_{in})^{k_i}) \quad (2)$$

$$\Delta_c = c_{out} - c_{in} \quad (3)$$

$$\alpha = \int_0^1 \Delta_c dc_{in} = \int_0^1 \left(\prod_i (1-(1-c_{in})^{k_i}) - c_{in} \right) dc_{in}, \quad (4)$$

where i is the metabolite index and k_i is the number of alternative sources for this metabolite. Reactions with empty sets of sources were removed from the analysis.

Then if $\alpha > 0$ node is regarded as OR, in the case of $\alpha < 0$ node is AND. The value of a obtained with this method is between 0.08 and 0.24 for human metabolic models (see Table 1). These values are clearly located in the subcritical regime. This is in line with our model results, which show that only the subcritical regime is viable and resilient to defect and perturbations.

We also checked metabolic models for other living organisms and we found substantial agreement between the values of a. This is a suitable starting point for further investigation. Also, the precise determination of this parameter deserves a more detailed study.

References

1. Duarte, N. C. *et al.* Global reconstruction of the human metabolic network based on genomic and bibliomic data. *PNAS* **104**, 1777–1782 (2007).
2. Thiele, I. *et al.* A community-driven global reconstruction of human metabolism. *Nature Biotechnology* **31**, 419–425 (2013).
3. Brunk, E. *et al.* Recon3D enables a three-dimensional view of gene variation in human metabolism. *Nature Biotechnology* **36**, 272–281 (2018).
