# Supplementary material for: Network location and clustering of genetic mutations determine chronicity in a stylized model of genetic diseases
Source: Sci Rep. 2022 Nov 19;12:19906. doi: 10.1038/s41598-022-23775-9 (PMC9675813; doi:10.1038/s41598-022-23775-9)
Supplement: Supplementary file 2 — Supplementary Figures. [file 41598_2022_23775_MOESM2_ESM.pdf]

Supplementary information: Network location and clustering of genetic mutations determine chronicity in a stylized model of genetic diseases

Piotr Nyczka[1,2], **Johannes Falk**[2,*], **and Marc-Thorsten Hütt**[2]

[1]Faculty of Management, Wrocław University of Science and Technology
[2]Department of Life Sciences and Chemistry, Jacobs University, D-28759 Bremen, Germany
[*]j.falk@jacobs-university.de

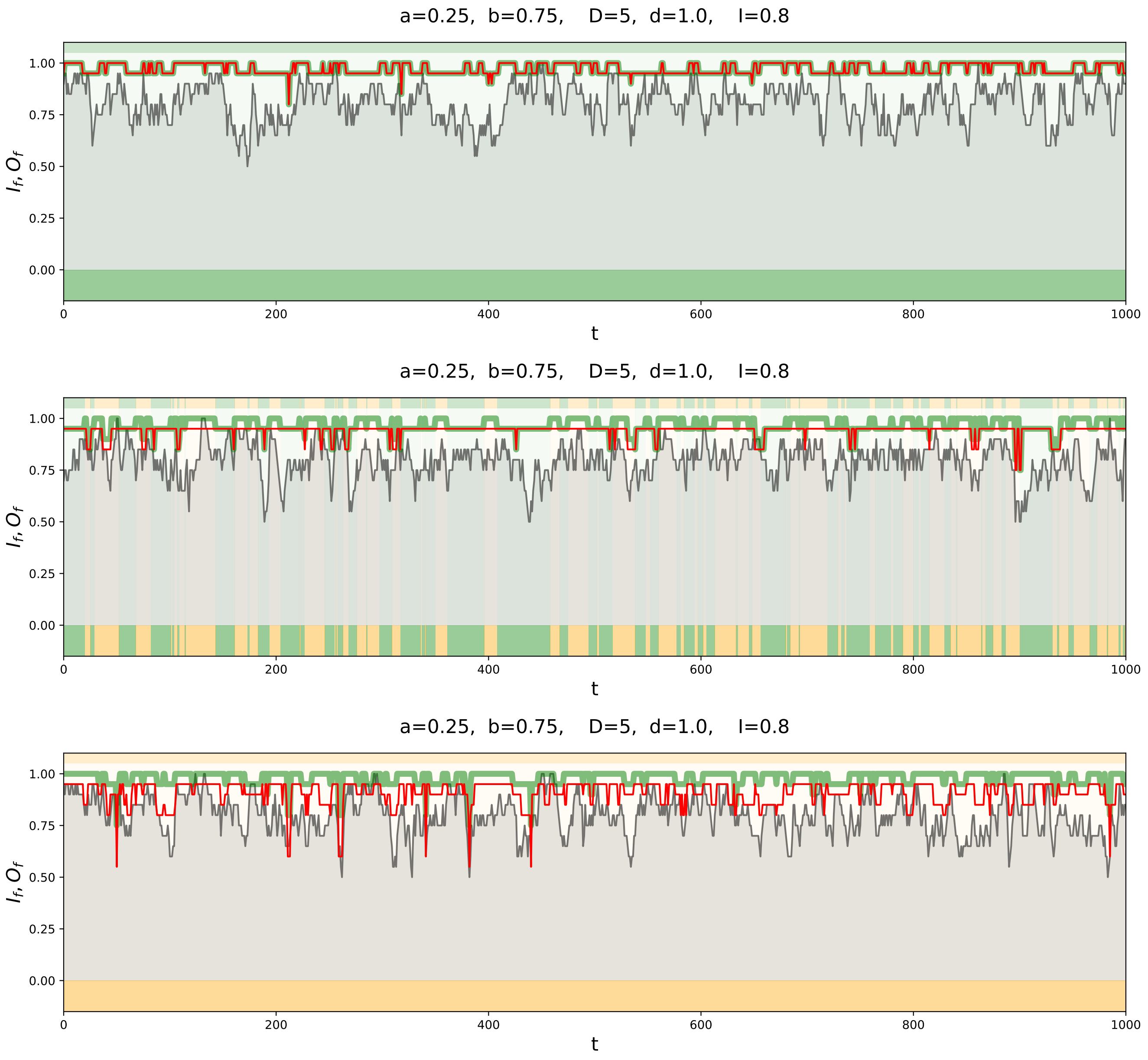

Figure S1. Three examples of possible time trajectories. From top to bottom: A, AB and B (colour code as defined in the main text). Note, that the model parameters are equal. Hence, the differences in the observed disease depend only on the particular realization. Other parameters: $L = 10, H = 20$.

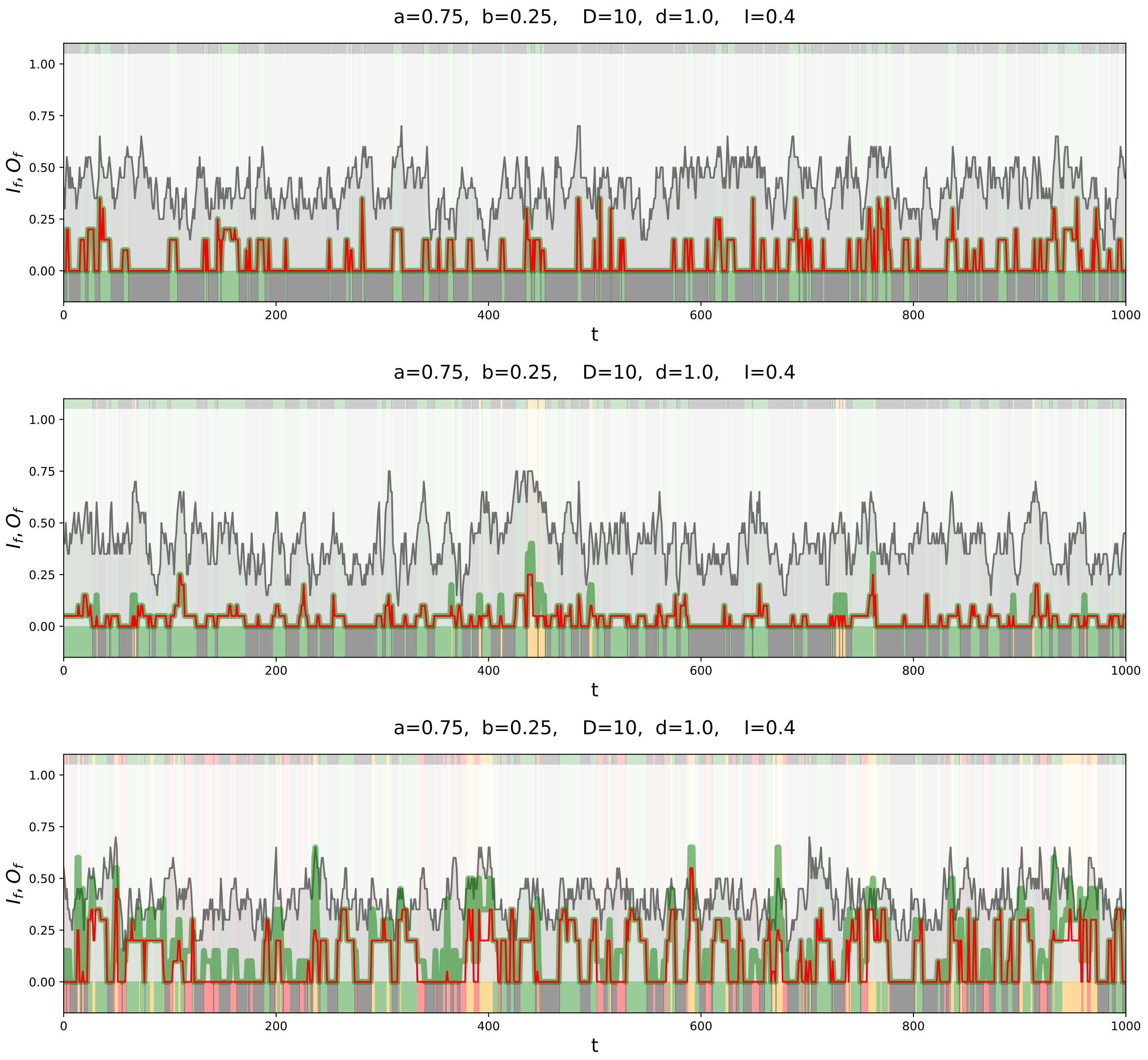

Figure S2. Three examples of possible time trajectories. From top to bottom: AD, ABD, ABCD (colour code as defined in the main text). Other parameters: $L = 20$, $H = 20$.

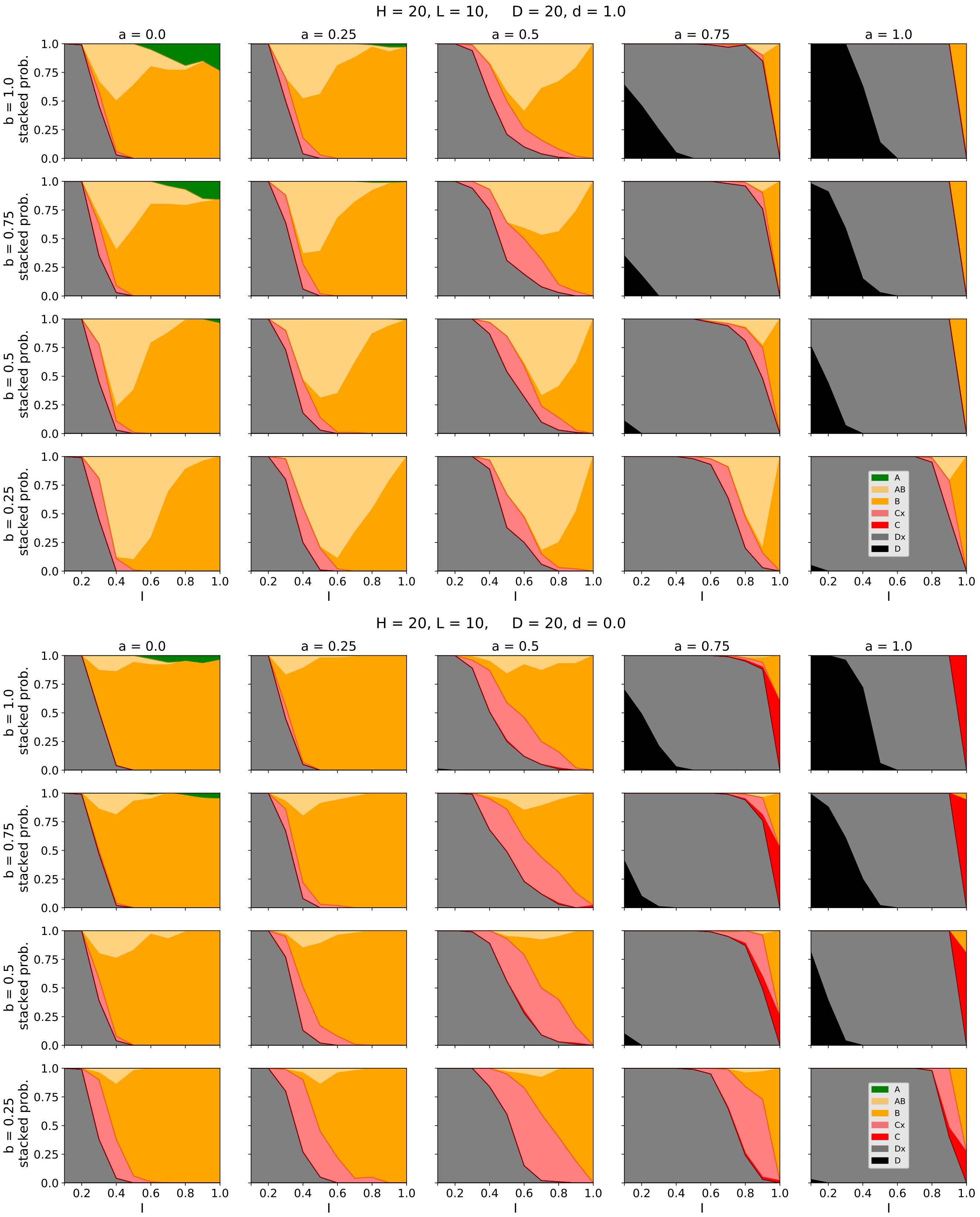

Figure S3. Dependence of the observed cases on the fraction of active inputs I. For each parameter combination, the system was simulated 100 times for 1000 time steps. The colour code (as defined in the main text) indicates which cases occurred in the time line.

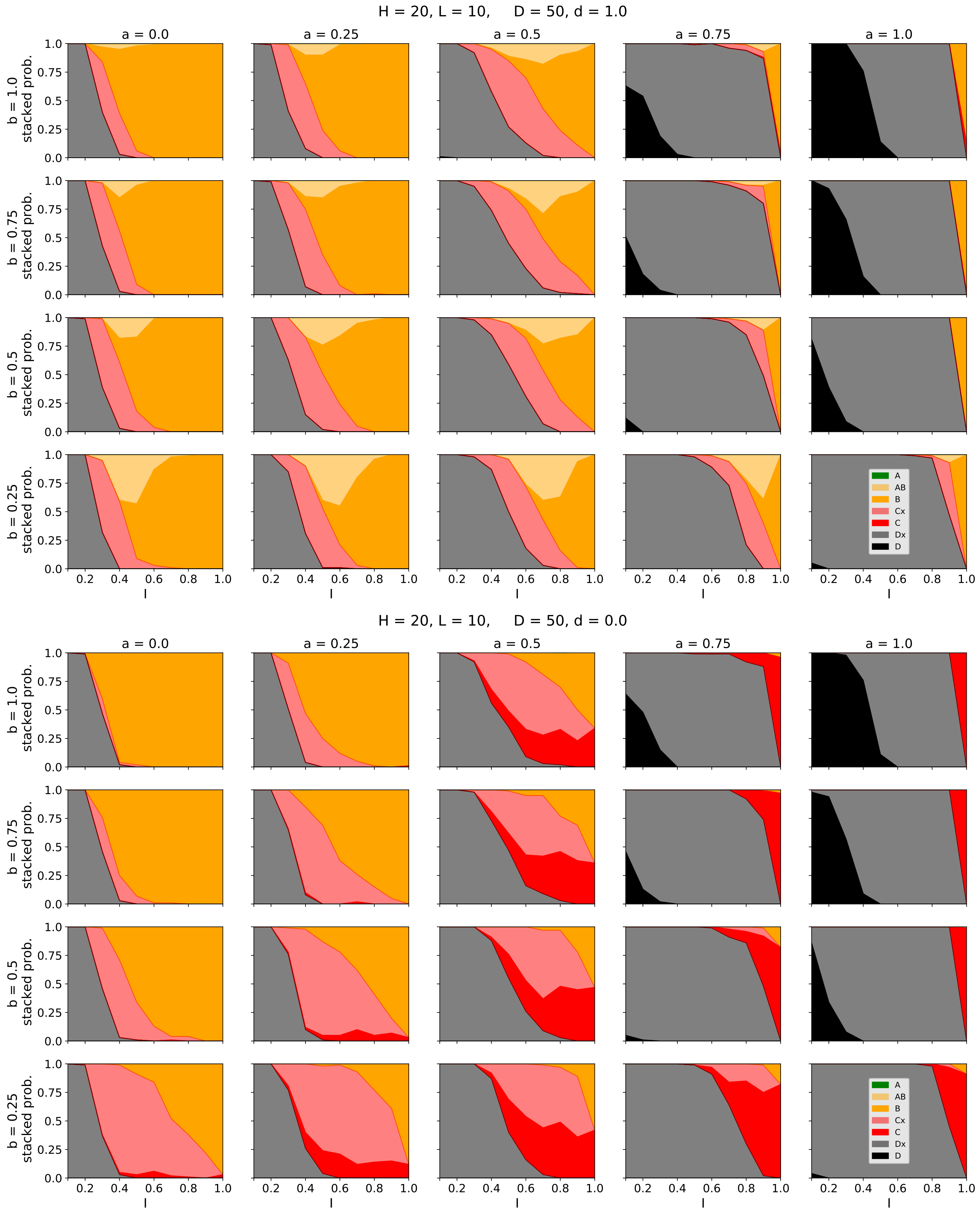

Figure S4. Dependence of the observed cases on the fraction of active inputs I. For each parameter combination, the system was simulated 100 times for 1000 time steps. The colour code (as defined in the main text) indicates which cases occurred in the time line.

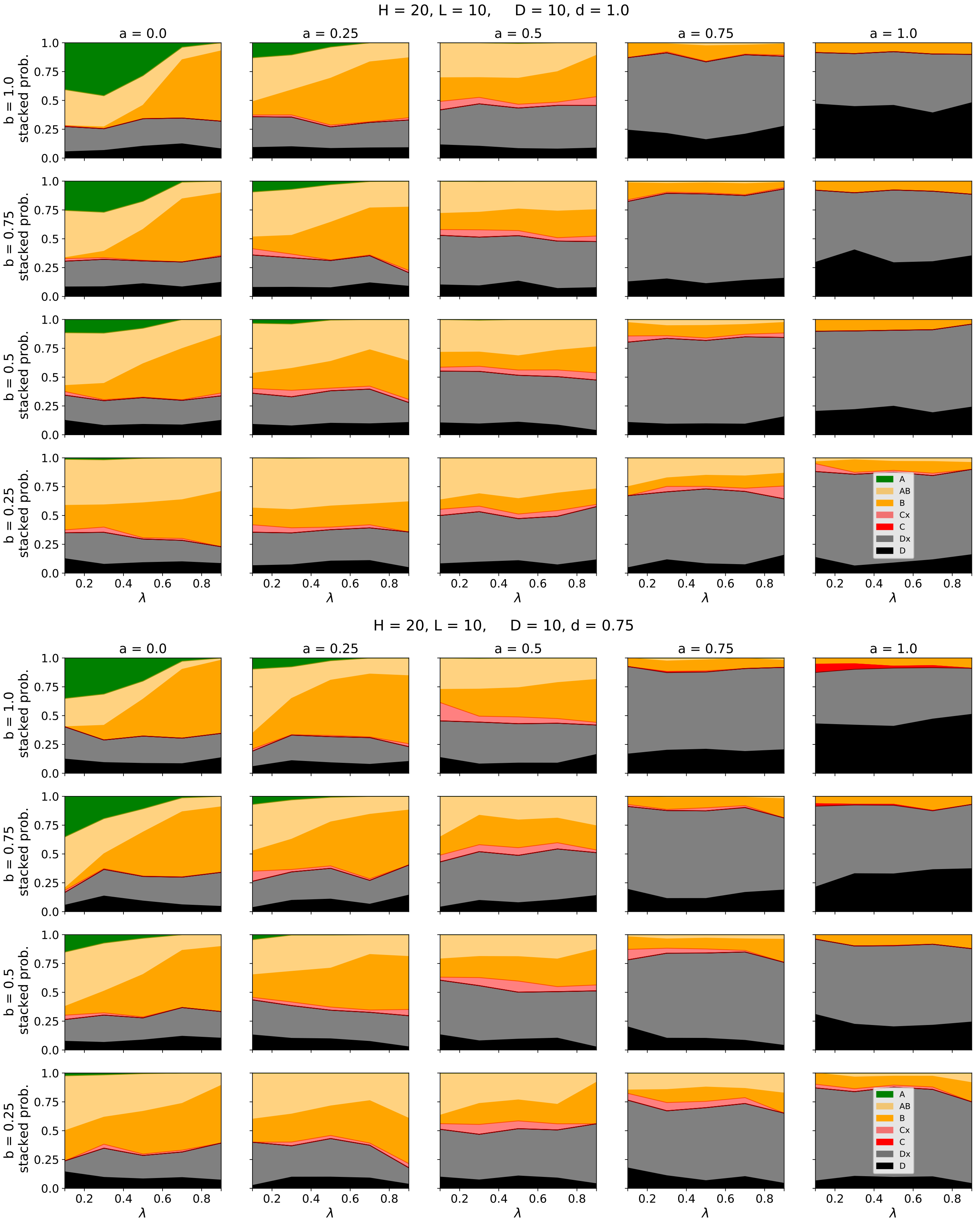

Figure S5. Dependence of the observed cases on the average position λ of inactive nodes. The colour code (as defined in the main text) indicates which cases occurred in the time line.

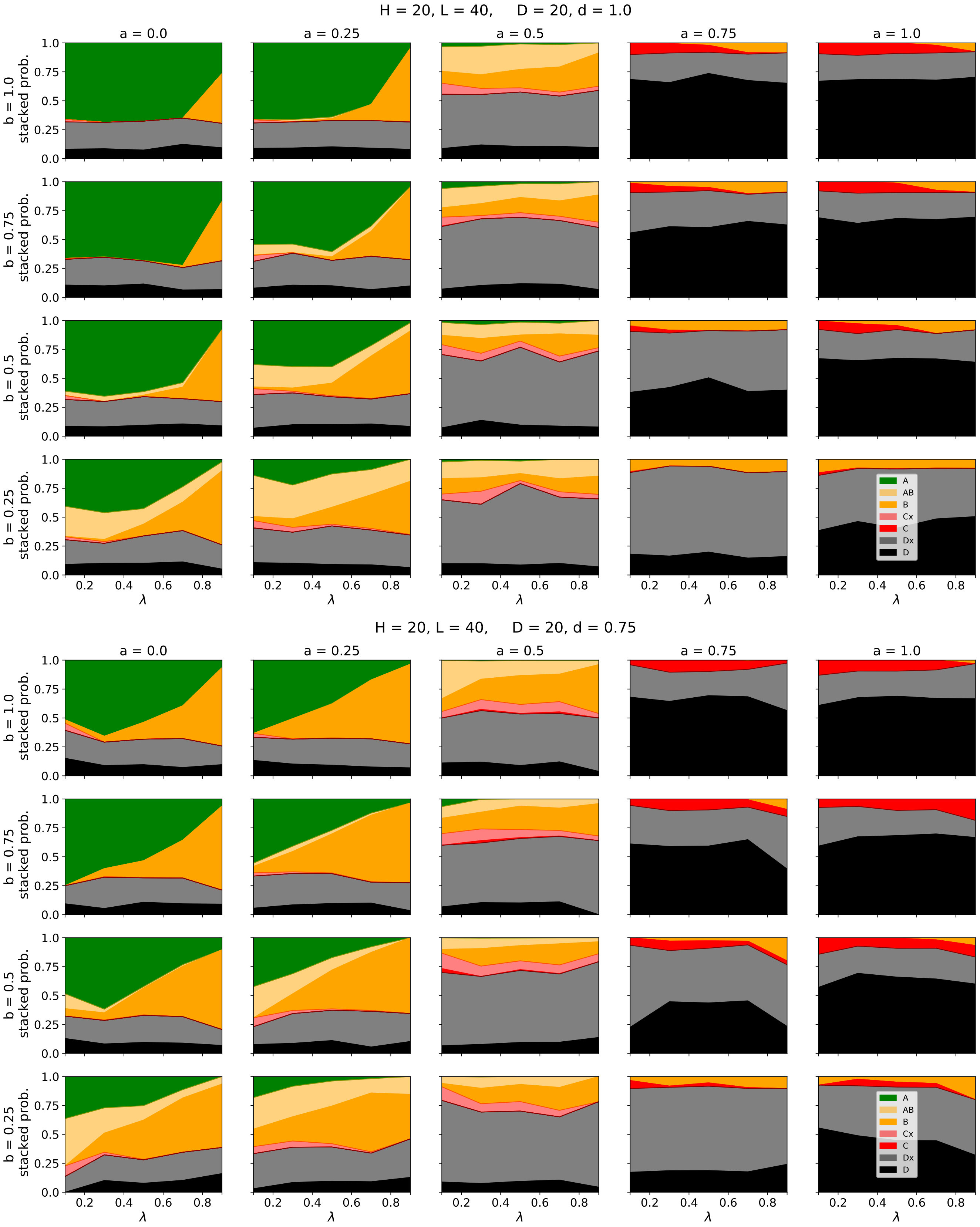

Figure S6. Dependence of the observed cases on the average position λ of inactive nodes. The colour code (as defined in the main text) indicates which cases occurred in the time line.
